# The Agreement Between Preoperative Digital Radiographic Measurements and Clinically Fitted Stainless Steel Crown Size in Primary Molars: A Retrospective Study

**DOI:** 10.3390/dj14090566

**Published:** 2026-09-04

**Authors:** Mayar Alharbi, Eman Bakhurji, Ashwin C. Shetty, Mohammed Alsaati, Yousef Alyousef

**Affiliations:** 1 Fellowship Program in Pediatric Dentistry, College of Dentistry, Imam Abdulrahman Bin Faisal University, P.O. Box 1982, Dammam 31441, Saudi Arabia; 2 Department of Preventive Dental Sciences, College of Dentistry, Imam Abdulrahman Bin Faisal University, P.O. Box 1982, Dammam 31441, Saudi Arabia; 3 Department of Dental Education, College of Dentistry, Imam Abdulrahman Bin Faisal University, P.O. Box 1982, Dammam 31441, Saudi Arabia; 4 Department of Maxillofacial and Oral Health, King Fahd Hospital of the University, Imam Abdulrahman Bin Faisal University, P.O. Box 1982, Dammam 31441, Saudi Arabia

**Keywords:** stainless steel crowns, crown size, digital radiographs, primary molars

## Abstract

**Background/Objectives:** Accurate selection of stainless steel crown (SSC) size is essential for successful restoration of primary molars. Conventional crown selection relies on a trial-and-error approach, which may increase chair time and reduce clinical efficiency. Preoperative digital radiographic measurements may provide an objective adjunct for predicting SSC size; however, evidence supporting their agreement with clinically selected crown sizes remains limited. This study aimed to evaluate the agreement between preoperative digital radiographic measurements and clinically fitted SSC size in primary molars, and to assess whether this agreement is influenced by dental arch or tooth type. **Methods:** This retrospective cross-sectional study analyzed archived patient records and preoperative digital radiographs of pediatric patients who received SSCs on primary molars. Mesiodistal tooth dimensions were measured and converted to SSC sizes according to manufacturer guidelines. The clinically fitted and cemented crown size, as recorded in the patient chart, served as the reference standard against which radiographically predicted size was compared. Intra-examiner reliability was assessed using the intraclass correlation coefficient. Associations were analyzed using chi-square tests and binomial logistic regression (*p* ≤ 0.05). Given repeated teeth within patients, the primary logistic regression model was additionally re-fit using generalized estimating equations (GEEs) with an exchangeable working correlation structure and robust standard errors, and prediction error was further characterized using near-miss accuracy, direction of bias, weighted kappa, and Bland–Altman analysis. **Results:** A total of 152 primary molars from 71 patients were included. Intra-examiner reliability was excellent (ICC = 0.99). Radiographic prediction agreed with the clinically fitted crown size in 33.6% of cases, and 69.7% of predictions fell within one crown size of the cemented crown. Agreement was significantly higher for mandibular molars than maxillary molars (*p* = 0.010) and varied by tooth type (*p* < 0.001). Mandibular molars were more likely to show accurate prediction (OR = 2.52; 95% CI: 1.23–5.17); this association remained statistically significant after accounting for within-patient clustering using GEEs with an exchangeable correlation structure (OR = 2.46; 95% CI: 1.10–5.49; *p* = 0.029). Maxillary first molars showed a systematic under-prediction of exactly two crown sizes in 97% of cases (mean bias −2.00), identifying a specific, correctable calibration problem. **Conclusions:** Preoperative digital radiographic measurements show moderate agreement with clinically fitted crown size and may serve as a useful adjunct to clinical judgment for SSC size selection, particularly for mandibular molars. Because the reference standard reflects clinical judgment itself rather than an independent anatomical gold standard, these findings should be interpreted as a concordance analysis, and further prospective work is needed before efficiency benefits can be confirmed.

## 1. Introduction

Dental caries remains the most prevalent chronic disease affecting children worldwide and continues to pose a major public health challenge despite advances in preventive and restorative dentistry [1,2]. Primary molars are particularly susceptible to carious destruction due to their morphological characteristics, thinner enamel, and prolonged functional lifespan in the oral cavity. If left untreated, caries in primary molars may result in pain, infection, premature tooth loss, and adverse effects on mastication, speech, and the eruption and alignment of permanent successors [3]. Consequently, the preservation of primary molars through durable and effective restorative interventions is a fundamental objective in pediatric dental care.

Among the available restorative options, SSCs are widely regarded as the treatment of choice for primary molars affected by extensive caries, multisurface lesions, or following pulp therapy [4,5]. SSCs provide full coronal coverage, protect the remaining tooth structure, and demonstrate superior longevity and success rates compared with intracoronal restorations such as amalgam, composite resin, or glass ionomer cement [6]. Numerous clinical and retrospective studies have consistently reported high survival rates for SSCs, regardless of whether they are placed using conventional preparation techniques or minimally invasive approaches such as the Hall technique [7,8,9]. Even when compared with newer esthetic alternatives, including prefabricated zirconia crowns, SSCs continue to demonstrate greater durability and lower rates of biological and mechanical complications, particularly in complex clinical cases [10,11,12].

Despite their well-established clinical advantages, the placement of SSCs presents several practical challenges, one of the most significant being accurate crown size selection. SSCs are prefabricated restorations available in standardized sizes, and optimal clinical outcomes depend on selecting the smallest crown that fully seats on the prepared tooth while maintaining proper marginal adaptation and proximal contacts [13,14]. This precision is not merely a matter of convenience: an improper crown size and a poor fit at the cervical margin are closely associated with invasion of the biological space, a technical complication that triggers immediate hyperemia of the gingival tissue, leading to persistent gingival inflammation and compromising the clinical longevity of the restoration. Traditionally, crown selection relies on a trial-and-error approach, during which multiple crowns may be tried before an acceptable fit is achieved. This process can be time-consuming, increase material waste, and prolong chair time, factors that are especially problematic in pediatric patients with limited cooperation or anxiety [15,16].

Efficiency is a critical consideration in pediatric dentistry, particularly when treating young children or patients managed under conscious sedation or general anesthesia. Prolonged operative time has been associated with increased behavioral challenges and may compromise treatment outcomes. Reducing the number of crown try-ins during SSC placement could therefore improve clinical efficiency, enhance patient comfort, and optimize workflow in both hospital-based and private practice settings [4,8]. Identifying methods that facilitate more accurate initial crown selection is thus of considerable clinical importance.

Radiographic assessment has long been employed in dentistry to estimate anatomical dimensions and guide treatment planning in disciplines such as endodontics, orthodontics, and implant dentistry. In pediatric dentistry, several investigations have demonstrated a relationship between the mesiodistal dimensions of primary molars and the dimensions of prefabricated SSCs, suggesting that objective measurements may assist in crown size selection [17,18]. Studies evaluating the compatibility between primary molar dimensions and SSCs have reported that anatomical variations exist between maxillary and mandibular arches, as well as between first and second primary molars, which may influence the accuracy of crown adaptation [19].

Advancements in digital radiography have further enhanced the potential utility of radiographic measurements by allowing for precise linear measurements with improved image quality and reproducibility. Recent studies have explored the dimensional correspondence between primary molars and SSCs using digital measurement tools, reporting variable but promising levels of agreement depending on tooth type and arch [18,20]. However, much of the existing literature has focused on measurements obtained from dental casts or extracted teeth, limiting the direct clinical applicability of these findings.

There remains limited evidence regarding the agreement between preoperative digital radiographic measurements obtained from routine clinical records and the SSC size ultimately selected in primary molars. Moreover, the influence of anatomical factors such as dental arch and tooth type on this agreement has not been adequately explored. Addressing these gaps could provide clinicians with a practical, noninvasive adjunct to clinical judgment, potentially reducing reliance on repeated crown trials during SSC placement. Therefore, the aim of this study was to assess the agreement between preoperative digital radiographic measurements and clinically fitted SSC size selection for primary molars using archived patient records and radiographs. In addition, the study sought to evaluate whether this agreement is influenced by dental arch or tooth type. It was hypothesized that radiographic measurements could serve as a reliable adjunct to conventional clinical judgment, thereby enhancing efficiency and supporting evidence-based decision-making in pediatric restorative dentistry.

## 2. Materials and Methods

### 2.1. Study Design and Ethical Approval

This study was designed as a retrospective cross-sectional investigation based on the review of archived patient records and preoperative digital radiographs. No direct patient contact, clinical intervention, or additional radiographic exposure occurred for the purpose of this study. Ethical approval was obtained from the Institutional Review Board of Imam Abdulrahman Bin Faisal University, Dammam, Saudi Arabia (IRB No. IRB-2024-02-368). This study is reported in accordance with the STROBE (Strengthening the Reporting of Observational Studies in Epidemiology) guidelines for cross-sectional studies. Full details of the informed consent waiver and data availability are provided in the Institutional Review Board Statement, Informed Consent Statement, and Data Availability Statement at the end of this manuscript.

### 2.2. Study Setting and Data Source

**Figure 1:**
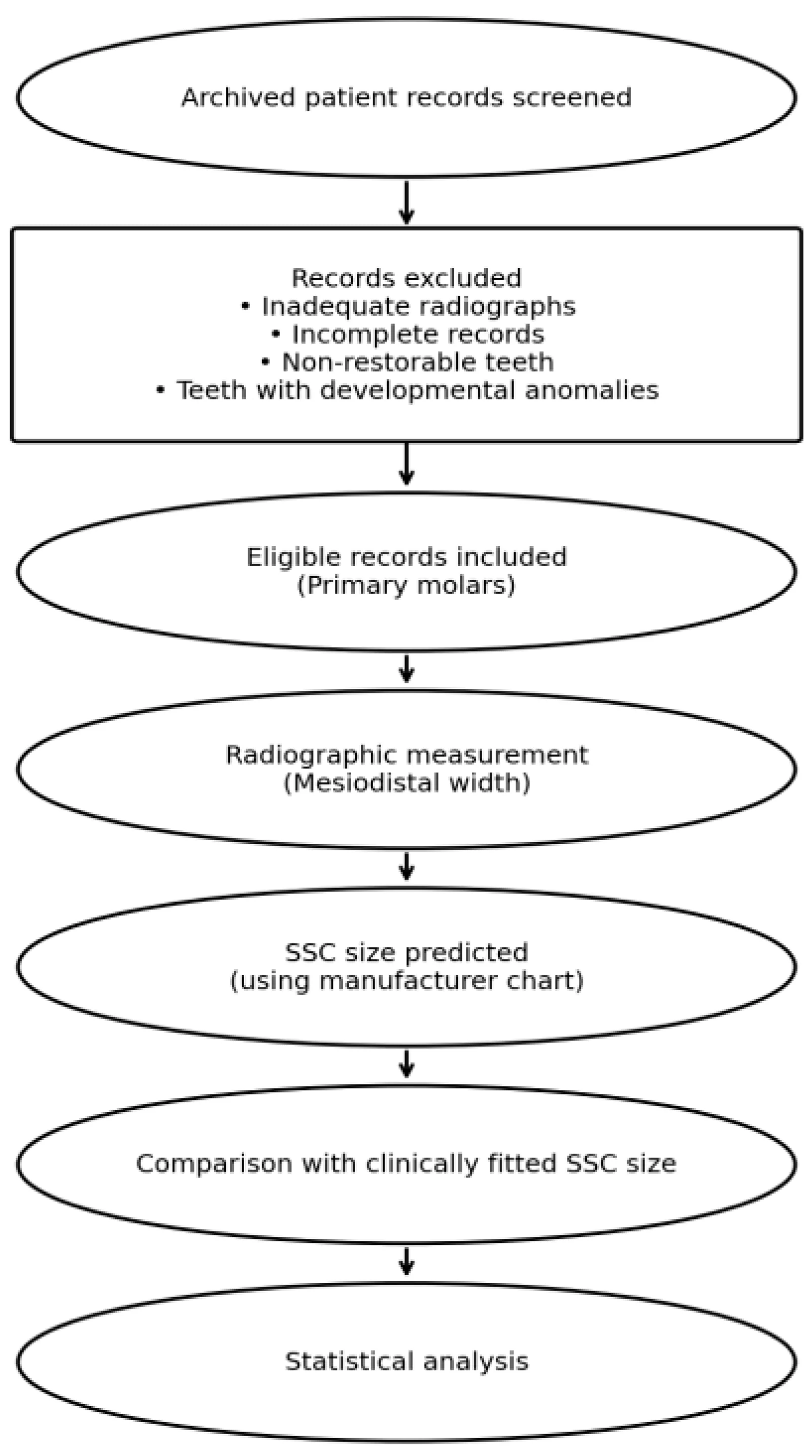
Flow diagram of archived record screening, exclusion, and final inclusion.

The study was conducted at the Pediatric Dentistry outpatient clinics, College of Dentistry, Imam Abdulrahman Bin Faisal University Dental Hospital, Dammam, Saudi Arabia. This retrospective study utilized archived electronic dental records and associated digital radiographs to identify pediatric cases in which stainless steel crowns (SSCs) had been placed on primary molars as part of routine dental care. Data extraction was performed following approval from the Institutional Review Board (IRB-2024-02-368). All data were obtained exclusively from existing, de-identified clinical records, and no direct patient contact or new data collection was undertaken. The overall study design and the process of archived record screening, exclusion, and final inclusion are illustrated in Figure 1.

### 2.3. Record Selection and Sample Size Calculation

Patient records were eligible for inclusion if they met the following criteria: children aged between 3 and 10 years who had received SSCs on maxillary or mandibular first or second primary molars, and for whom preoperative digital bitewing or periapical radiographs of diagnostic quality were available. Teeth included in the analysis demonstrated intact mesial and distal contact points, allowing for reliable mesiodistal measurement. Records were excluded if the treated teeth were deemed non-restorable, exhibited more than 2 mm of space loss due to extensive proximal caries, showed significant structural loss requiring substantial crown modification prior to cementation, or if the available radiographs were distorted or inadequate for accurate measurement. Teeth with developmental anomalies that could compromise measurement accuracy were also excluded (Figure 1).

The minimum required sample size was calculated for the primary study outcome, the proportion of primary molars for which radiographic measurement would accurately predict the clinically fitted SSC size. Because this outcome is a proportion to be estimated rather than a between-group comparison to be tested, sample size was calculated using the standard formula for estimating a single proportion within a specified margin of error at a given confidence level, *n* = Z^2^p(1 − p)/d^2^, where Z is the standard normal deviate corresponding to the desired confidence level, p is the expected proportion, and d is the acceptable margin of error; a formal power/beta term does not apply to this precision-based calculation, as statistical power is a property of hypothesis tests rather than interval estimation. The expected proportion was set at *p* = 0.40, consistent with the first-attempt crown size prediction accuracy rates of 38–46 percent reported for coronal and cervical radiographic length measurements by Helder et al. [20], the key prior study on which the present design was based. Using a 95% confidence level (Z = 1.96, corresponding to α = 0.05) and a margin of error of 10 percentage points, this yielded a minimum required sample size of 93 primary molars (*n* = 1.96^2^ × 0.40 × 0.60/0.10^2^ = 92.2, rounded up to 93). The final sample comprised 152 primary molars from 71 patients (an average of approximately 2.1 molars per patient), therefore exceeding this minimum requirement.

### 2.4. Radiographic Assessment and Measurement Protocol

Preoperative digital radiographs were retrieved and analyzed using MiPACS^®^ Dental Enterprise Viewer (Medicor Imaging, Charlotte, NC, USA). All measurements were performed on calibrated digital images to minimize measurement error. For each primary molar, the mesiodistal dimension was assessed using a standardized protocol adapted from previously published radiographic measurement methodologies [21,22]. Two linear measurements were obtained for each tooth: the maximum mesiodistal distance measured between the most convex points of the mesial and distal proximal surfaces, and a second mesiodistal measurement aligned parallel to the occlusal plane. The mean of these two measurements was calculated to represent the final mesiodistal tooth width. This value was subsequently converted into the corresponding stainless steel crown (SSC) size using the manufacturer’s sizing chart provided for prefabricated crowns (Hu-Friedy™, Chicago, IL, USA); a single manufacturer’s chart was used consistently for all cases to avoid introducing sizing convention discrepancies. All measurements were performed by a single calibrated examiner who was blinded to the clinically cemented crown size and clinical chart at the time of measurement; measurement records were linked only by study ID, and the examiner did not have access to the fitted crown size until all measurements and size conversions had been completed and locked in order to minimize the risk of measurement bias.

### 2.5. Reliability Assessment

To evaluate intra-examiner reliability, a randomly selected subset of 10 radiographs were measured by the same examiner on two separate occasions two weeks apart, under identical conditions. Reliability was assessed using the intraclass correlation coefficient (ICC), a statistical method widely used to assess measurement consistency in dental research [23]. An ICC value greater than 0.90 was considered indicative of excellent reliability. Because measurements were performed by a single calibrated examiner, inter-examiner reliability could not be assessed in this study; this is noted as a limitation in Section 4. High measurement reliability reflects the reproducibility of the radiographic measurement technique and does not, by itself, establish the predictive validity of that technique for crown size selection.

### 2.6. Statistical Analysis

The primary outcome variable was the agreement between the crown size predicted from radiographic measurements and the crown size documented as clinically fitted and cemented according to the patient record; agreement outcomes were categorized as accurate or inaccurate. Secondary variables included patient age, treatment setting (clinic, nitrous oxide sedation, or general anesthesia), dental arch (maxillary or mandibular), tooth position (first or second primary molar), and Back-to-Back contact, defined as the presence of direct mesial and/or distal proximal contact between the crown and the adjacent tooth, as documented in the clinical record (present vs. absent). Statistical analysis was performed using IBM SPSS Statistics for Windows (version 31.0). Descriptive statistics were used to summarize the distribution of cases according to demographic and clinical variables. The association between prediction accuracy and categorical variables, including treatment setting, dental arch, Back-to-Back contact, and tooth type, was assessed using chi-square tests. Variables demonstrating potential associations were further evaluated using binomial logistic regression analysis to identify predictors of accurate radiographic crown size selection. Model fit was assessed using the Hosmer–Lemeshow goodness-of-fit test, and odds ratios with 95% confidence intervals were calculated. Statistical significance was set at a *p*-value of less than 0.05.

Because the 152 study teeth were drawn from 71 patients (mean 2.14 teeth/patient), the primary logistic regression model was additionally re-fit using generalized estimating equations (GEEs) with an exchangeable working correlation structure, estimated via the method-of-moments alternating algorithm of Liang and Zeger, together with empirical (robust, sandwich) standard errors clustered on patient identifier and a small-sample correction. A design-effect sensitivity analysis (DEFF = 1 + (m − 1)ρ, where m is the mean cluster size) was additionally used to bound the potential impact of clustering across a plausible range of within-patient intraclass correlations (ρ).

To characterize prediction error beyond a binary accurate/inaccurate classification, we additionally calculated (i) the proportion of predictions falling within one crown size of the cemented crown; (ii) the signed difference (predicted–cemented size, in ordinal crown size units) to assess the direction of any systematic bias, overall and by tooth type; (iii) unweighted, linear-weighted, and quadratic-weighted Cohen’s kappa to quantify chance-corrected agreement while giving partial credit for near misses; and (iv) a Bland–Altman analysis (mean bias and 95% limits of agreement) comparing predicted and cemented crown size.

## 3. Results

A total of 152 primary molars restored with SSCs, drawn from 71 patients, were included in the analysis (an average of approximately 2.1 molars per patient). The mean age of the sample was 5.81 ± 1.81 years.

All cases had complete archived patient records and diagnostically acceptable preoperative digital radiographs, with no missing data identified. The distribution of cases according to patient age, treatment setting, dental arch, back-to-back contact, and tooth type is summarized in Table 1. More than half of the SSCs were placed under general anesthesia (51.3%), while the remaining cases were treated in outpatient clinic settings (25.0%) or with nitrous oxide sedation (23.7%). The distribution of teeth was evenly divided between the maxillary and mandibular arches (50.0% each). Back-to-back contact was present in 61.8% of cases, whereas 38.2% of teeth did not have adjacent crowns. With respect to tooth type, mandibular second primary molars constituted the largest proportion of the sample (30.3%), followed by maxillary second primary molars (29.6%), maxillary first molars (20.4%), and mandibular first molars (19.7%).

### 3.1. Association Between Radiographic Prediction Accuracy and Clinical/Anatomical Variables

Radiographic prediction of SSC size was accurate in 51 cases (33.6%) and inaccurate in 101 cases (66.4%). Table 2 shows the association between prediction accuracy and treatment setting, dental arch, Back-to-Back contact, and tooth type. Accurate prediction was observed in 28.9% of cases treated in the clinic, 38.9% of cases treated under nitrous oxide sedation, and 33.3% of cases treated under general anesthesia. No statistically significant association was found between treatment setting and prediction accuracy (χ^2^ = 0.823, df = 2, *p* = 0.663).

**Figure 2:**
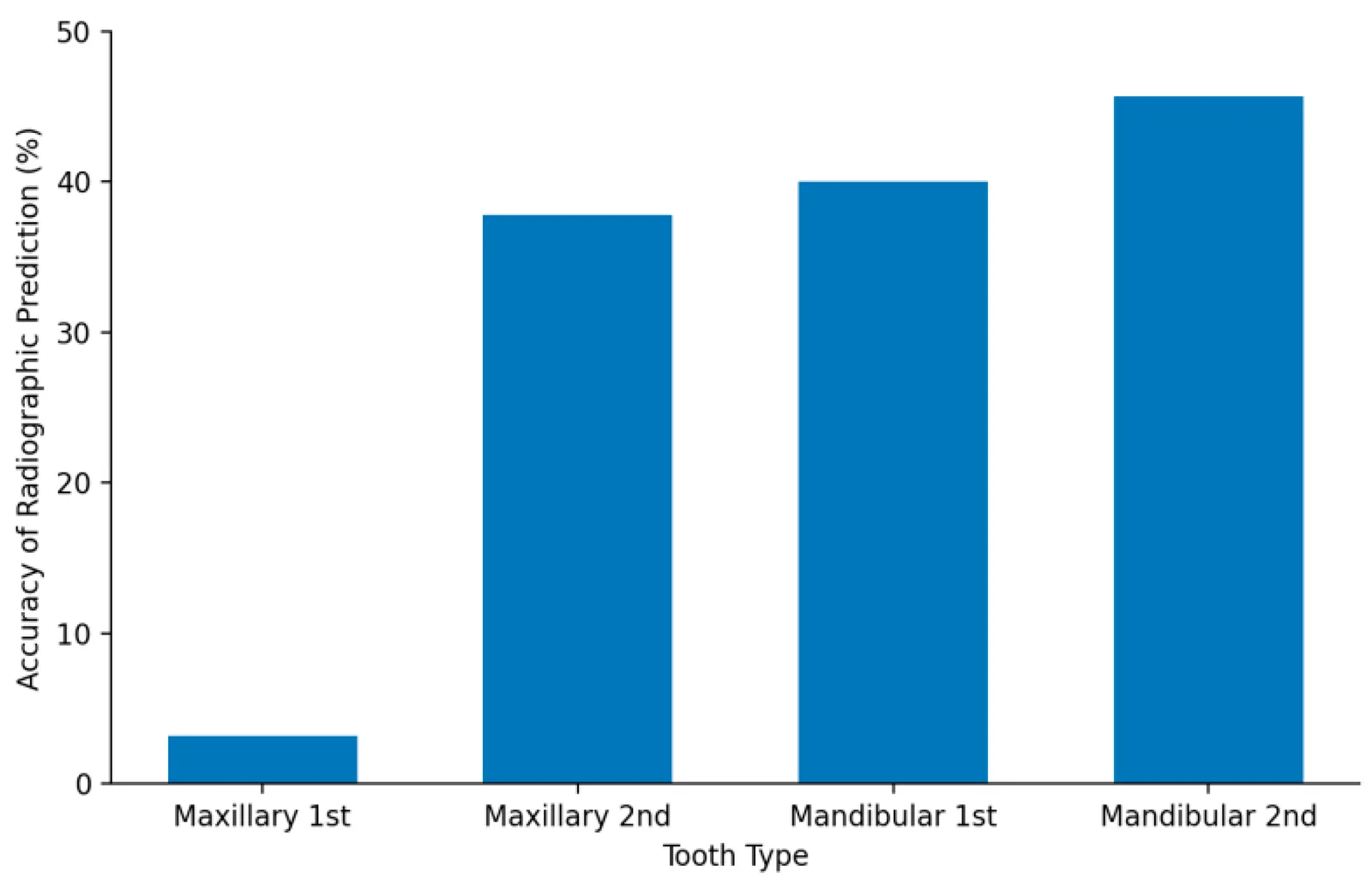
Accuracy of radiographic crown size prediction according to tooth type.

A statistically significant association was identified between prediction accuracy and dental arch. Accurate prediction occurred in 23.7% of maxillary molars compared with 43.4% of mandibular molars (χ^2^ = 6.639, df = 1, *p* = 0.010). Prediction accuracy was also assessed in relation to Back-to-Back contact. Accurate predictions were recorded in 29.8% of teeth with back-to-back contact and 39.7% of teeth without back-to-back contact. This difference was not statistically significant (χ^2^ = 1.567, df = 1, *p* = 0.211). A statistically significant association was observed between prediction accuracy and tooth type (χ^2^ = 16.729, df = 3, *p* < 0.001). Accurate prediction was lowest for maxillary first primary molars (3.2%) and progressively higher for maxillary second primary molars (37.8%), mandibular first primary molars (40.0%), and mandibular second primary molars (45.7%). These distributions are illustrated in Figure 2.

### 3.2. Logistic Regression Analysis

Binomial logistic regression analysis was conducted to identify predictors of accurate radiographic crown size prediction. The model included age, treatment setting, dental arch, and Back-to-Back contact as independent variables. Tooth type, which was the strongest univariate predictor of accuracy (*p* < 0.001), was not entered into the multivariable model because it is nested within, and collinear with, dental arch; dental arch was retained as the higher-order anatomical variable in this model. With only 51 accurate prediction events, the events-per-variable ratio for the four-predictor model is close to the commonly recommended minimum of ten, and these regression findings should therefore be interpreted as exploratory rather than confirmatory. The model demonstrated acceptable goodness of fit, with a non-significant Hosmer–Lemeshow test (χ^2^ = 8.570, df = 8, *p* = 0.380). The regression results are presented in Table 3. Dental arch was the only statistically significant predictor of accurate prediction. Mandibular molars were associated with higher odds of correct radiographic crown size prediction compared with maxillary molars (odds ratio = 2.52, 95% CI: 1.23–5.17; *p* = 0.012). Age, treatment setting, and back-to-back contact were not significantly associated with prediction accuracy (*p* > 0.05).

**Figure 3:**
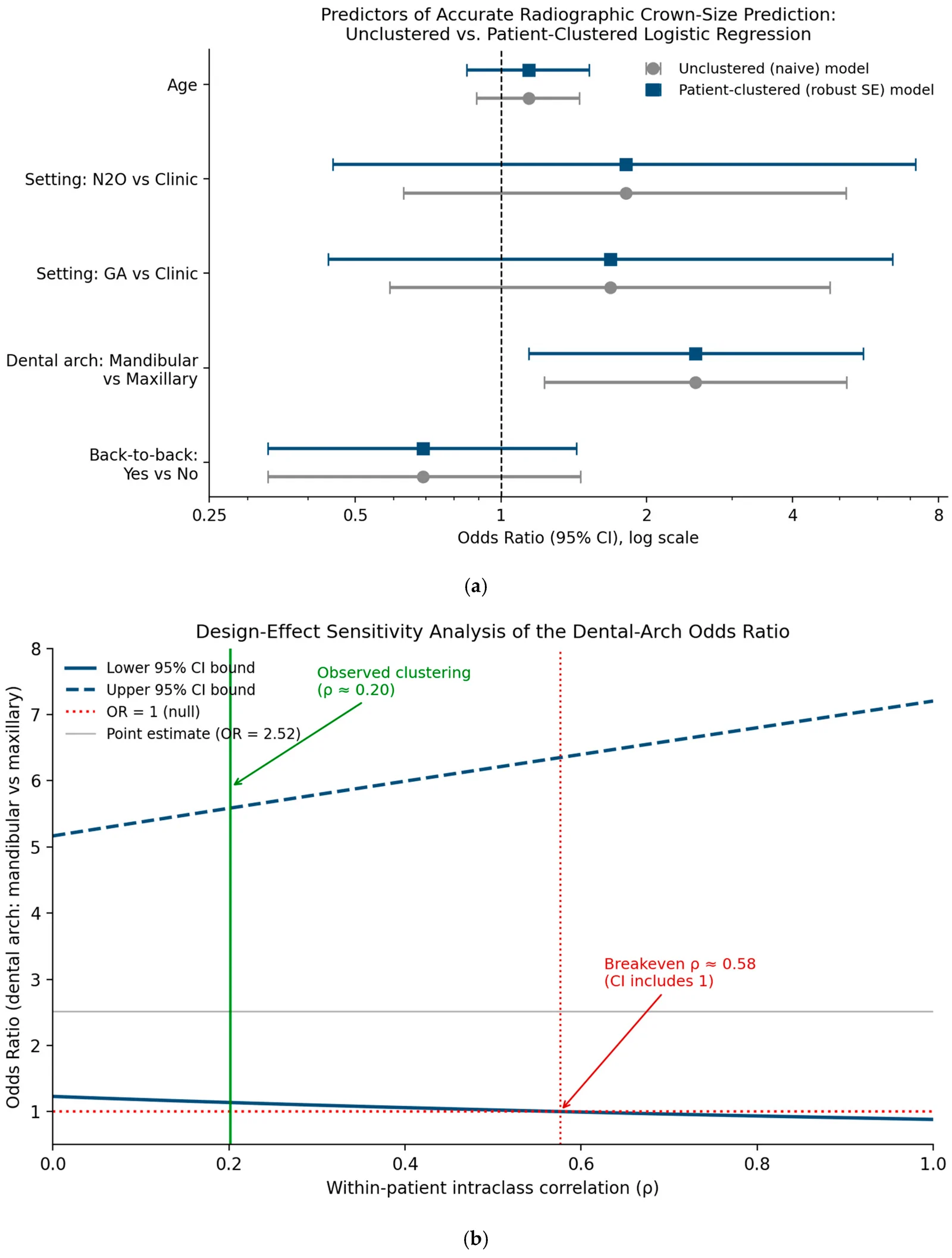
(**a**) A forest plot comparing unclustered and patient-clustered (GEE, exchangeable correlation) odds ratios (95% CI) for all model predictors. (**b**) Design-effect sensitivity analysis of the dental arch odds ratio across a range of plausible within-patient intraclass correlations (ρ), with the breakeven point and the empirically observed clustering level indicated.

To confirm this association was not an artifact of within-patient clustering, a design-effect sensitivity analysis was first performed using the reported odds ratio and observed mean cluster size (m = 2.14 teeth/patient). The 95% CI for the dental arch effect remained bounded away from the null for all within-patient intraclass correlations up to ρ ≈ 0.58, a level well beyond that typically observed for binary tooth-level outcomes in the dental literature. The model was then re-fit using GEEs with an exchangeable working correlation structure and robust standard errors (estimated working correlation α = 0.113). The point estimate for dental arch attenuated slightly (OR = 2.46) and the 95% CI widened to 1.10–5.49 (*p* = 0.029); the association remained statistically significant. Full results for all predictors under the clustering-adjusted model are shown in Table 4 and Figure 3.

### 3.3. Prediction Error, Direction of Bias, and Agreement

Because a binary accurate/inaccurate classification does not distinguish a near miss from a large discrepancy, prediction error was further characterized in ordinal crown size units (predicted size minus cemented size). Overall, 106 of 152 predictions (69.7%) fell within one crown size of the cemented crown, compared with the 33.6% exact-match rate reported above. The mean signed bias was −0.23 sizes (SD 1.42), reflecting a slight overall tendency toward under-prediction; 58 teeth (38.2%) were under-predicted, 43 (28.3%) were over-predicted, and 51 (33.6%) matched exactly. Chance-corrected agreement was substantial to near-perfect once near misses were given partial credit: unweighted κ = 0.256, linear-weighted κ = 0.679, and quadratic-weighted κ = 0.878.

Breaking these results down by tooth type (Table 5) revealed a clear, systematic pattern. Maxillary first primary molars—the tooth type with the lowest exact-match rate (3.2%, Section 3.1)—were under-predicted in 30 of 31 cases (97%), with a mean bias of exactly −2.00 crown sizes and no instances of over-prediction. In contrast, mandibular molars showed a milder lean toward over-prediction, most pronounced for mandibular second molars (54% over-predicted, 0% under-predicted). This directional asymmetry, rather than random measurement scatter, is consistent with a correctable offset in the size conversion step specific to maxillary first molars.

## 4. Discussion

This study assessed the agreement between preoperative digital radiographic measurements and clinically fitted crown size as an adjunct for SSC size selection in primary molars, using archived patient records and radiographs. The findings indicate that radiographic prediction accuracy was moderate overall, with correct crown size estimation achieved in approximately one-third of cases. However, prediction accuracy was significantly influenced by anatomical variables, particularly dental arch and tooth type, supporting the selective clinical use of radiographic estimation as an adjunct rather than its routine or exclusive application, and underscoring that these findings represent agreement with clinical selection rather than validation against an independent anatomical standard.

The overall prediction accuracy observed in this study is consistent with previous investigations that have demonstrated variable correspondence between primary molar dimensions and prefabricated SSC sizes [17,18,19,20,24]. Morphometric studies comparing tooth dimensions with SSCs have consistently reported that standardized crown sizes cannot perfectly accommodate the natural anatomical variability of primary molars, particularly in different populations [17,18,19]. These findings support the notion that radiographic measurements should be viewed as an adjunctive tool to guide initial crown selection rather than a definitive method for crown sizing.

A key finding of the present study was the significantly higher accuracy of radiographic prediction for mandibular molars compared with maxillary molars. This result aligns with multiple previous studies reporting better dimensional compatibility and measurement reliability in mandibular primary molars [19,21,23,24]. Anatomical factors such as reduced superimposition of adjacent structures, more favorable proximal contact geometry, and clearer visualization of crown contours in mandibular molars may contribute to this improved accuracy [21,22]. In contrast, maxillary molars are more susceptible to projection errors and overlapping anatomical structures, which can compromise measurement precision on two-dimensional radiographs [22].

Tooth type was also found to significantly influence prediction accuracy, with mandibular second primary molars demonstrating the highest accuracy and maxillary first primary molars the lowest. Previous morphometric and clinical studies have reported greater variability in the crown morphology of maxillary first primary molars, which may limit the reliability of radiographic estimation for this tooth type [17,18,23]. Conversely, mandibular second primary molars tend to exhibit more consistent mesiodistal dimensions, resulting in improved correspondence with prefabricated SSCs [23,24].

The particularly low accuracy observed for maxillary first primary molars (3.2%) is unlikely to reflect random measurement variation alone and may instead indicate a systematic error, such as an off-by-one discrepancy in the size conversion chart applied to this tooth type.

**Figure 4:**
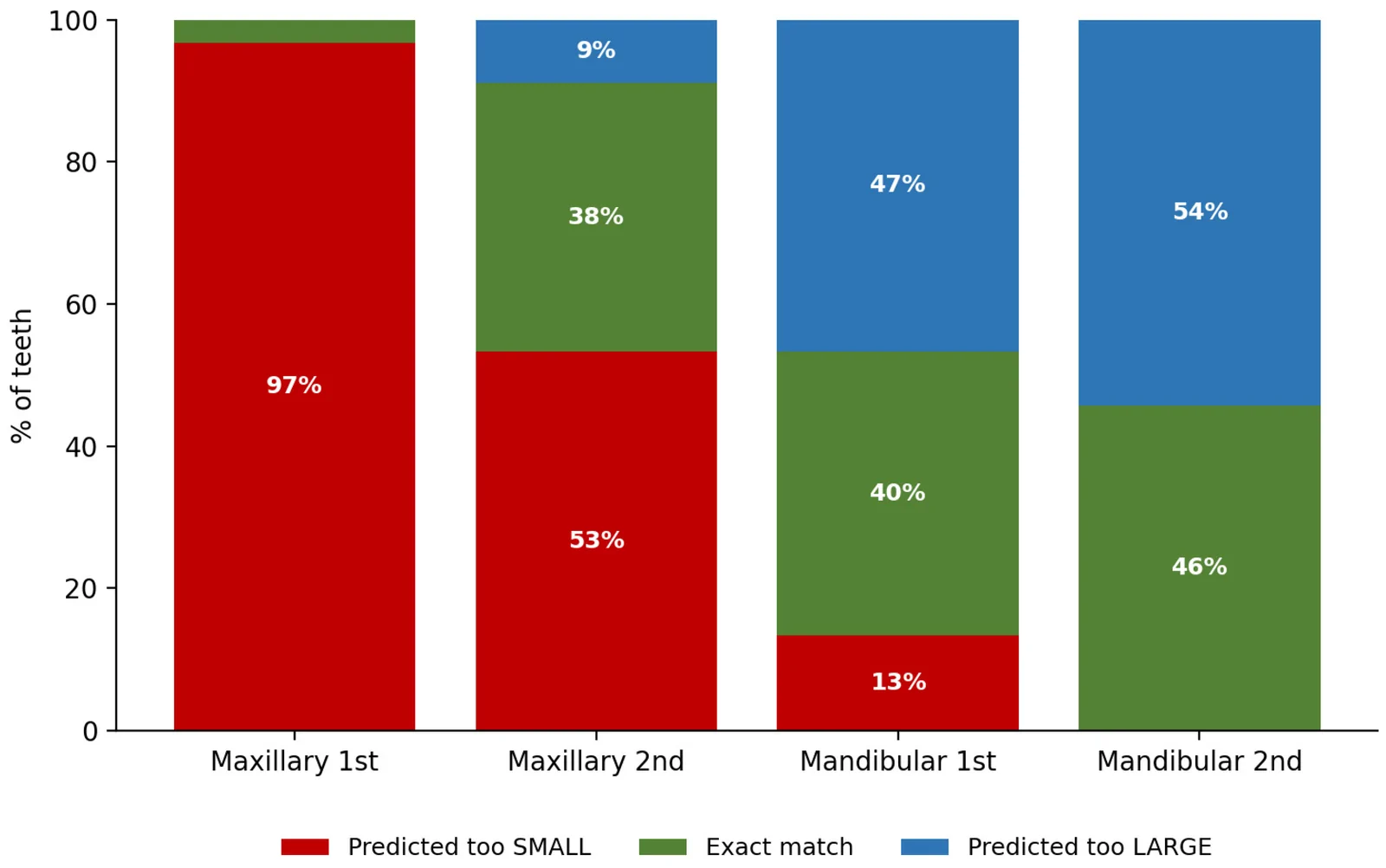
Direction of radiographic prediction error by tooth type.

**Figure 5:**
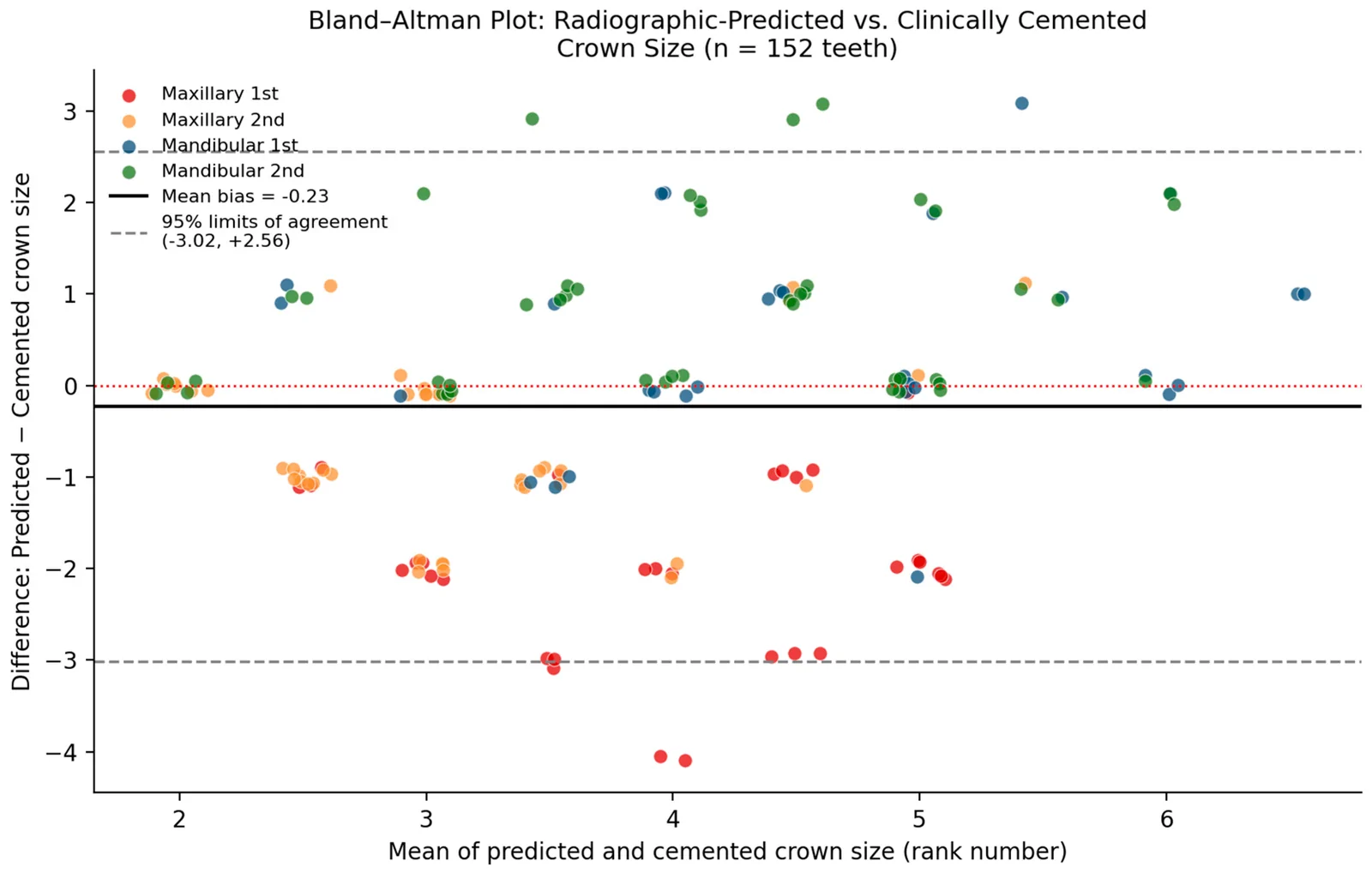
Bland–Altman plot of radiographic-predicted vs. clinically cemented crown size (mean bias −0.23; 95% limits of agreement −3.02 to +2.56), colored by tooth type.

This has now been confirmed quantitatively: 30 of 31 maxillary first molars (97%) were under-predicted, with a mean bias of exactly −2.00 crown sizes and zero instances of over-prediction (Section 3.3, Table 5, Figure 4 and Figure 5). The near-total uniformity of both the direction and magnitude of this error—rather than a symmetric scatter around zero—indicates a systematic, correctable offset specific to maxillary first molars, most plausibly in the radiographic-to-crown size conversion step for this tooth type, rather than genuine anatomical unpredictability. This represents a directly actionable finding: applying a fixed +2-size correction for maxillary first-molar predictions, and re-validating this correction prospectively, could substantially improve the clinical utility of radiographic estimation for this tooth type specifically.

The lack of a significant association between prediction accuracy and treatment setting suggests that radiographic estimation may be applied consistently across different clinical contexts, including routine clinical care, nitrous oxide sedation, and general anesthesia. This finding is clinically relevant, as SSCs are frequently placed under general anesthesia, where efficiency and reduction in operative time are critical considerations [1,15]. The ability to narrow crown size selection prior to treatment may plausibly reduce the number of crown try-ins required; however, as noted below, this study did not directly measure chair time, try-ins, or related outcomes, and this potential benefit remains to be confirmed.

Although Back-to-Back contact did not significantly affect prediction accuracy, this finding is consistent with previous reports indicating that proximal contact status alone does not reliably predict crown adaptation or measurement precision [14]. Similarly, patient age was not a significant predictor of accuracy, suggesting that radiographic estimation may be applicable across the pediatric age range studied, provided that radiographic quality and tooth integrity are adequate.

From a clinical standpoint, the findings of this study support the targeted use of preoperative digital radiographic measurements as a supportive tool in SSC size selection, particularly for mandibular and second primary molars. This approach aligns with contemporary principles of evidence-based pediatric dentistry, which emphasize efficiency, minimally invasive decision-making, and reduction in unnecessary procedural steps [7,25,26]. Importantly, radiographic estimation should not replace clinical judgment or trial fitting, but rather assist clinicians in making more informed initial crown selections. It should also be emphasized that this study did not directly measure chair time, number of crown try-ins, material waste, or clinical outcomes; statements regarding improved efficiency should therefore be regarded as a hypothesis for future prospective research rather than a demonstrated finding of the present study.

Several limitations should be considered when interpreting the results of this study.

First, the reference standard used to judge radiographic “accuracy” was the clinically fitted and cemented crown size, itself the product of trial-and-error clinical judgment; this study therefore reflects agreement between radiographic estimation and eventual clinical selection rather than validation against an independent anatomical gold standard, and the framing and title of this manuscript have been revised accordingly to describe an agreement/concordance analysis.

Second, the retrospective design relied on archived patient records and radiographs, which may be subject to variability in radiographic angulation, magnification, and image quality [21,22,27]. This concern is particularly relevant for preschool-aged children (approximately three to four years old), for whom obtaining high-quality periapical radiographs is diagnostically challenging: dental anxiety and a pronounced gag reflex often make it difficult for the child to remain completely still, resulting in motion artifacts and unacceptable image blurring, while the shallow floor of the mouth and low palatal vault characteristic of the primary dentition can hinder ideal placement of rigid sensors or film holders. Clinicians managing these patients often resort to modified radiographic techniques that introduce projection errors such as elongation, shortening, or interproximal overlap, which can compromise clear visualization of the furcation area and root apices and, by extension, the clarity of the mesiodistal measurements on which this study’s predictions depend. Third, the two-dimensional nature of conventional radiographs limits the ability to capture the three-dimensional morphology of primary molars, which may affect measurement accuracy [22,28].

Fourth, the 152 study teeth were drawn from 71 patients (an average of approximately 2.1 molars per patient), so some children contributed multiple teeth to the analysis. Because multiple molars from the same child are likely to be correlated, the independence assumption underlying the chi-square tests and logistic regression may be violated, which could bias standard errors, confidence intervals, and *p*-values, including the reported dental arch odds ratio.

This concern was directly evaluated using a GEE model with an exchangeable working correlation structure (α = 0.113) and robust standard errors, fitted to the raw tooth-level data. The dental arch odds ratio was materially unchanged (OR = 2.46 vs. 2.52) and remained statistically significant after clustering (95% CI: 1.10–5.49, *p* = 0.029). This is no longer regarded as an unresolved limitation of the primary finding.

Fifth, prediction accuracy was scored as a binary correct/incorrect exact match. This approach does not distinguish a prediction that was off by a single crown size from one that was substantially discrepant, nor does it indicate the direction of error.

This has now been addressed (Section 3.3): 69.7% of predictions fell within one crown size of the cemented crown; the method showed a slight overall under-prediction bias (mean −0.23 sizes) that was strongly tooth type-dependent; weighted kappa statistics (linear = 0.679, quadratic = 0.878) confirmed substantial-to-near-perfect chance-corrected agreement once near misses were credited; and a Bland–Altman analysis (Figure 5) quantified the bias and 95% limits of agreement (−3.02 to +2.56 sizes).

Sixth, a single calibrated examiner performed all measurements, limiting generalizability, and inter-examiner reliability was not assessed.

Seventh, this study’s inclusion criteria required intact mesial and distal contact points for reliable mesiodistal measurement, and teeth with substantial structural loss or non-restorable status were excluded (Section 2.3). A completely destroyed primary tooth with pulp pathology at the coronal level, in which the mesio-distal reference landmarks have been lost, represents one of the most complex scenarios in pediatric dentistry, introducing anatomical, clinical, and biomechanical limitations that clinicians must document with particular rigor. In the absence of these landmarks, clinicians tend to underestimate or overestimate tooth diameter; if an oversized crown is selected, marginal overcontouring can occur, invading the interproximal space, damaging the gingival papilla, and altering contact points with adjacent teeth. Because such cases were excluded from the present analysis, the reported agreement rates should not be extrapolated to grossly destroyed teeth lacking mesio-distal landmarks; radiographic estimation may be least reliable, and potentially most valuable, in precisely this subgroup, and this represents an important direction for future study.

Finally, the full distribution of patient age and the exact numbers of records screened and excluded at each stage were not completely reported and could not be included in this analysis.

Future studies should consider prospective designs with standardized radiographic protocols, blinded assessors, statistical models that account for clustering of multiple teeth within the same patient, and the inclusion of multiple examiners to assess inter-examiner reliability. The use of three-dimensional imaging modalities or digital intraoral scanning technologies may further improve preoperative crown size prediction [28,29]. Additionally, the development of tooth-specific or population-specific sizing algorithms may enhance the clinical applicability of radiographic estimation techniques.

## 5. Conclusions

Preoperative digital radiographic measurements can serve as a useful adjunct to clinical judgment in the selection of SSC sizes for primary molars. While overall agreement with clinically fitted crown size was moderate, radiographic estimation demonstrated significantly higher agreement for mandibular molars and specific tooth types. These findings suggest that incorporating radiographic measurements into clinical decision-making may help guide initial crown selection and could potentially reduce the need for multiple crown trials; however, because chair time, number of try-ins, and clinical outcomes were not directly measured in this study, these potential efficiency benefits should be regarded as hypotheses to be tested in future prospective research rather than demonstrated findings. Because the reference standard for “accurate” prediction was itself derived from clinical judgment, these findings should be interpreted as reflecting agreement between radiographic estimation and clinical selection rather than independent validation against a true anatomical gold standard. Radiographic prediction should not replace conventional clinical assessment but may provide valuable support, particularly in pediatric patients and treatment settings where minimizing chair time is a goal for future research to address.

The clustering-adjusted (GEE, exchangeable correlation) and near-miss/bias analyses reported in Section 3.2 and Section 3.3 confirm that the dental arch association is robust to within-patient correlation and identify a specific, quantifiable, and potentially correctable calibration offset for maxillary first primary molars, providing a concrete direction for improving the method’s clinical utility in future prospective work.

## Figures and Tables

**Table 1:** Distribution of study sample according to demographic and clinical variables (*n* = 152).

Variable	Category	*n* (%)
Treatment setting	Clinic	38 (25.0)
	Nitrous oxide	36 (23.7)
	General anesthesia	78 (51.3)
Dental arch	Maxillary	76 (50.0)
	Mandibular	76 (50.0)
Back-to-Back Contact	Yes	94 (61.8)
	No	58 (38.2)
Tooth type	Maxillary first molar	31 (20.4)
	Maxillary second molar	45 (29.6)
	Mandibular first molar	30 (19.7)
	Mandibular second molar	46 (30.3)

**Table 2:** Prediction accuracy by treatment setting, dental arch, back-to-back contact, and tooth type.

Variable	Category	Accurate *n* (%)	Inaccurate *n* (%)	*p*-Value
Treatment setting	Clinic	11 (28.9)	27 (71.1)	0.663
	Nitrous oxide	14 (38.9)	22 (61.1)	
	General anesthesia	26 (33.3)	52 (66.7)	
Dental arch	Maxillary	18 (23.7)	58 (76.3)	0.010
	Mandibular	33 (43.4)	43 (56.6)	
Back-to-Back Contact	Yes	28 (29.8)	66 (70.2)	0.211
	No	23 (39.7)	35 (60.3)	
Tooth type	Maxillary first	1 (3.2)	30 (96.8)	<0.001
	Maxillary second	17 (37.8)	28 (62.2)	
	Mandibular first	12 (40.0)	18 (60.0)	
	Mandibular second	21 (45.7)	25 (54.3)	

**Table 3:** Logistic regression analysis predicting accurate radiographic crown size selection (unclustered model).

Variable	Odds Ratio (Exp B)	95% CI	*p*-Value
Age	1.14	0.90–1.45	0.289
Treatment setting	—	—	0.507
Dental arch (mandibular)	2.52	1.23–5.17	0.012
Back-to-Back Contact	0.69	0.33–1.46	0.334

**Table 4:** Dental arch odds ratio and other predictors: unclustered vs. GEE exchangeable correlation model.

Variable	Model	OR	95% CI	*p*-Value
**Dental arch (Mandibular vs. Maxillary)**	Unclustered (original)	2.52	1.23–5.17	0.012
	GEE, exchangeable correlation (definitive)	**2.46**	**1.10–5.49**	**0.029**
Age	GEE, exchangeable correlation	1.16	0.87–1.53	0.311
Setting: N2O vs. Clinic	GEE, exchangeable correlation	1.87	0.49–7.06	0.357
Setting: GA vs. Clinic	GEE, exchangeable correlation	1.72	0.48–6.16	0.407
Back-to-back: Yes vs. No	GEE, exchangeable correlation	0.72	0.34–1.51	0.383

**Table 5:** Near-miss accuracy and direction of prediction error by tooth type.

Tooth Type	*n*	Exact Match	Within 1 Size	Mean Bias	Predicted Too Small	Exact	Predicted Too Large
**Maxillary 1st**	31	3.2%	29.0%	**−2.00**	**30 (97%)**	1	0
Maxillary 2nd	45	37.8%	84.4%	−0.60	24 (53%)	17	4
Mandibular 1st	30	40.0%	80.0%	+0.50	4 (13%)	12	14 (47%)
Mandibular 2nd	46	45.7%	76.1%	+0.85	0 (0%)	21	**25 (54%)**
**Overall**	**152**	**33.6%**	**69.7%**	**−0.23**	**58 (38%)**	**51**	**43 (28%)**

## Data Availability

De-identified participant data supporting the findings of this study are available from the corresponding author upon reasonable request and subject to approval by the Institutional Review Board and the data-sharing policies of Imam Abdulrahman Bin Faisal University. Due to privacy and ethical restrictions, patient-level data are not publicly deposited.

## References

[1] Ramazani N. (2017). Stainless steel crowns in pediatric restorative dentistry: Applications and advantages. Innov. J. Pediatr..

[2] Nowak A.J., Christensen J.R., Mabry T.R., Townsend J.A., Wells M.A. (2018). Pediatric Dentistry: Infancy Through Adolescence.

[3] Waggoner W.F. (2002). Restoring primary anterior teeth: Updated guidelines for the clinician. Pediatr. Dent..

[4] Threlfall A.G., Pilkington L., Milsom K.M., Blinkhorn A.S., Tickle M. (2005). General dental practitioners’ views on the use of stainless steel crowns to restore primary molars. Br. Dent. J..

[5] Seale N.S., Randall R. (2015). The use of stainless steel crowns: A systematic literature review. Pediatr. Dent..

[6] Wu E., Yang Y.J., Munz S.M., Hsiao C.C., Boynton J.R. (2021). Restorations versus stainless steel crowns in primary molars: A retrospective split-mouth study. Pediatr. Dent..

[7] Ludwig K.H., Fontana M., Vinson L.A., Platt J.A., Dean J.A. (2014). The success of stainless steel crowns placed with the Hall technique: A retrospective study. J. Am. Dent. Assoc..

[8] Sharaf D., Dowidar K., El Habashy L., Hamed H. (2021). Hall technique versus the conventional stainless steel crowns restoring carious primary molar teeth: A randomized controlled clinical trial. Alex. Dent. J..

[9] Elbahary S., Aharonian S., Azem H., Peretz B., Mostinski O., Blumer S. (2023). Bacterial colonization and proliferation in primary molars following the use of the Hall technique: A confocal laser scanning microscopy study. Children.

[10] Ram D., Sherman M., Polak D., Davidovich E. (2025). Radicular pathology in zirconia and stainless-steel crowns in primary posterior teeth: A retrospective comparative longitudinal study. Children.

[11] Kara N.B., Yilmaz Y. (2014). Assessment of oral hygiene and periodontal health around posterior primary molars after their restoration with various crown types. Int. J. Paediatr. Dent..

[12] Lopez-Cazaux S., Aiem E., Velly A.M., Muller-Bolla M. (2019). Preformed pediatric zirconia crown versus preformed pediatric metal crown: Study protocol for a randomized clinical trial. Trials.

[13] Zareiyan M., Molaasadolah F., Haghgoo R., Ahmadi R., Kahvand M. (2020). Reconstruction of pulpotomized primary molar and retention of stainless-steel crowns: An in-vitro study. Open Dent. J..

[14] Subramaniam P., Kondae S., Gupta K.K. (2010). Retentive strength of luting cements for stainless steel crowns: An in vitro study. J. Clin. Pediatr. Dent..

[15] Klingberg G., Broberg A.G. (2007). Dental fear/anxiety and dental behaviour management problems in children and adolescents: A review of prevalence and concomitant psychological factors. Int. J. Paediatr. Dent..

[16] American Academy of Pediatric Dentistry (2022). Behavior guidance for the pediatric dental patient. The Reference Manual of Pediatric Dentistry.

[17] Afshar H., Kamali Sabeti A., Shahrabi M. (2015). Comparison of primary molar crown dimensions with stainless steel crowns in a sample of Iranian children. J. Dent. Res. Dent. Clin. Dent. Prospects.

[18] Afshar H., Ghandehari M., Soleimani B. (2015). Comparison of marginal circumference of two different pre-crimped stainless steel crowns for primary molars after re-crimping. J. Dent..

[19] Hajiahmadi M., Zamani S., Mokarram M., Soltanian M. (2025). Comparison of the morphological dimensions of primary molars with kids stainless steel crowns in a sample of Iranian children. J. Contemp. Dent. Pract..

[20] Helder C., Alimorad L., Bodt B. (2022). Stainless steel crown size selection predicted by digital radiographic primary molar measurements. Pediatr. Dent..

[21] Farman A.G., Farman T.T. (1998). Panoramic dental radiography using a charge-coupled device receptor. J. Digit. Imaging.

[22] Setzer F.C., Lee S.M. (2021). Radiology in endodontics. Dent. Clin. N. Am..

[23] Moorrees C.F., Thomsen S.O., Jensen E., Yen P.K. (1957). Mesiodistal crown diameters of the deciduous and permanent teeth. J. Dent. Res..

[24] Shahrabi M., Heidari A., Kamareh S. (2019). Comparison of primary mandibular first molar crown dimensions with stainless steel crowns in a sample of Iranian children. Front. Dent..

[25] Altoukhi D.H., El-Housseiny A. (2020). Hall technique for carious primary molars: A review of the literature. Dent. J..

[26] American Academy of Pediatric Dentistry (2022). Pediatric restorative dentistry. The Reference Manual of Pediatric Dentistry.

[27] White S.C., Pharoah M.J. (2014). Oral Radiology: Principles and Interpretation.

[28] Mangano F., Gandolfi A., Luongo G., Logozzo S. (2017). Intraoral scanners in dentistry: A review of the current literature. BMC Oral Health.

[29] Ender A., Mehl A. (2013). Accuracy of complete-arch dental impressions: A new method of measuring trueness and precision. J. Prosthet. Dent..

